# The Histone Demethylase HR Suppresses Breast Cancer Development through Enhanced CELF2 Tumor Suppressor Activity

**DOI:** 10.3390/cancers14194648

**Published:** 2022-09-24

**Authors:** Yao Shen, Jasvinder Singh, Bindeshwar Sah, Zhongming Chen, Wootae Ha, Christine Henzler, Tao Su, Lillian Xie, Yibin Deng, Gen Li, Hua Guo, Hanina Hibshoosh, Liang Liu

**Affiliations:** 1 Department of Systems Biology, Columbia University Irving Medical Center, New York, NY 10032, USA; 2 The Hormel Institute, University of Minnesota, Austin, MN 55912, USA; 3 Minnesota Supercomputing Institute, University of Minnesota, Minneapolis, MN 55455, USA; 4 Department of Pathology, Columbia University Irving Medical Center, New York, NY 10032, USA; 5 Department of Urology, University of Minnesota Medical School, Minneapolis, MN 55455, USA; 6 Masonic Cancer Center, University of Minnesota, Minneapolis, MN 55455, USA; 7 Department of Biostatistics, School of Public Health, University of Michigan, Ann Arbor, MI 48109, USA

**Keywords:** hairless, breast cancer, histone methylation, CELF2, epigenetic therapy

## Abstract

**Simple Summary:**

We performed targeted sequencing to identify somatic mutations at the hairless (HR) gene locus in human breast tumors. We found HR mutations in approximately 15% of the patient cohort (n = 85), compared with 23% for BRCA2, 13% for GATA3, 7% for BRCA1, and 3% for PTEN in the same breast cancer patient cohort. We also found an average 23% HR copy number loss in breast cancers. HR reconstitution in HR-deficient human breast cancer cells significantly suppressed tumor growth in orthotopic xenograft mouse models. We further demonstrated that HR’s antitumor activity was at least partly mediated by transcriptional activation of CELF2, a tumor suppressor with RNA-binding activity. Finally, we showed that pharmacologic inhibition of histone methylation is effective in suppressing HR-deficient breast tumor growth and progression. These findings suggest that HR loss plays an important role in breast cancer pathogenesis and will lead to future studies to explore specific inhibitors of HR-dependent histone methylation pathway to abrogate HR-deficient tumor growth and progression.

**Abstract:**

The *hairless* (*HR*) gene encodes a transcription factor with histone demethylase activity that is essential for development and tissue homeostasis. Previous studies suggest that mutational inactivation of HR promotes tumorigenesis. To investigate HR mutations in breast cancer, we performed targeted next-generation sequencing using DNA isolated from primary breast cancer tissues. We identified *HR* somatic mutations in approximately 15% of the patient cohort (n = 85), compared with 23% for *BRCA2*, 13% for *GATA3*, 7% for *BRCA1*, and 3% for *PTEN* in the same patient cohort. We also found an average 23% *HR* copy number loss in breast cancers. In support of HR’s antitumor functions, HR reconstitution in *HR*-deficient human breast cancer cells significantly suppressed tumor growth in orthotopic xenograft mouse models. We further demonstrated that HR’s antitumor activity was at least partly mediated by transcriptional activation of *CELF2*, a tumor suppressor with RNA-binding activity. Consistent with HR’s histone demethylase activity, pharmacologic inhibition of histone methylation suppressed HR-deficient breast cancer cell proliferation, migration and tumor growth. Taken together, we identified HR as a novel tumor suppressor that is frequently mutated in breast cancer. We also showed that pharmacologic inhibition of histone methylation is effective in suppressing HR-deficient breast tumor growth and progression.

## 1. Introduction

Breast cancer (BC) is the most commonly diagnosed malignancy (excluding non-melanoma skin cancer) and the second leading cause of cancer-related death among women in the United States [1]. It is a heterogeneous disease, encompassing various subtypes with distinct origins, behaviors, prognoses, and responses to therapy [2]. Cancer genomic studies have revealed high-risk predisposing germline mutations, such as *BRCA1*/*BRCA2*, and other medium- to low-risk mutations in familial BC cases, including *CHEK2*, *TP53*, *PTEN*, and *CDH1* [3,4]. Despite decades of intense research into the genetic mechanisms involved in the initiation and progression of BC, only about 10% of cases are linked to pathogenic alterations in known risk genes [3]. The causes of the majority of breast malignancies remain elusive. It has now been established that the initiation and progression of BC, traditionally considered a genetic disease, involve epigenetic abnormalities in conjunction to genetic alterations [5]. Epigenetic regulators, such as DNA methyltransferases and histone-modifying enzymes, play essential roles in gene regulation and cancer development [6,7,8,9]. Genetic alterations affecting the function or enzymatic activity of these epigenetic regulators have been linked to cancer and other developmental disorders [8,10,11]. Histone methyltransferase dysregulation, for example, has been causally linked with BC pathogenesis [11,12,13,14].

The *hairless* (*HR*) gene encodes a transcription factor that is essential for development and tissue homeostasis [15,16]. Major functional domains of the HR protein are conserved between humans, rats, and mice, including a zinc finger domain, multiple LXXLL motifs, which mediate protein–protein interactions, and a Jumonji C (JmjC) domain at the C-terminus [14]. Previous studies have demonstrated that the role of HR in transcriptional regulation is partly mediated by binding to the thyroid hormone receptor, retinoic-acid-receptor-related orphan receptors, or the vitamin D receptor [14,17]. The JmjC domain is a signature motif among histone demethylases [8,18]. We recently reported that HR is a histone demethylase that targets mono- or di-methylated histone H3 lysine 9 (H3K9me1 or me2) [19]. Whereas H3K9me1 is associated with both expressed and repressed genes, H3K9me2 is generally associated with repressed genes and heterochromatin [8,13,20]. By demethylating H3K9me1 or me2, HR may epigenetically suppress or activate gene expression via chromatin remodeling. 

Mice with loss-of-function *Hr* mutations are hairless and susceptible to UV-induced skin tumorigenesis [21], which is suggestive of HR’s antitumor function in skin tumorigenesis. We previously identified several HR target genes that are important regulators of cell proliferation and apoptosis [19,22,23,24,25,26]. Analysis of the cBioPortal for Cancer Genomics database revealed over 200 missense *HR* mutations in various human cancer types [14,27]. Notably, mutations affecting HR JmjC domain are reported in BC [14]. Herein, we provide evidence showing *HR* mutations are highly prevalent in breast tumors based on targeted next-generation sequencing. In vitro and xenograft mouse experiments further supported the antitumor function of HR that is at least partly mediated via CELF2 in BC growth and progression. We also show that inhibition of H3K9 methylation abrogates the growth and migration of HR-deficient BC cells. 

## 2. Materials and Methods

### 2.1. Cell Culture and Transfection

Human BC cell lines (ATCC) were cultured in RPMI1640 medium supplemented with 10% fetal bovine serum and 1% penicillin-streptomycin (Life Technologies, Carlsbad, CA, USA). All cell lines were grown in a humidified atmosphere with 5% CO_2_ at 37 °C, authenticated, and tested for mycoplasma infection (Lonza, Rockland, ME, USA). *HR*-expressing and control lentiviral vectors were designed and prepared by Vector Builder (VectorBuilder Inc., Chicago, IL, USA). *CELF2*-expressing and control DNA plasmids were obtained from Addgene (#96900). Cell transfection was performed using the jetOPTIMUS^®^ reagent (Polyplus-transfection^®^, Illkirch-Graffenstaden, France). Following lentiviral transduction or plasmid DNA transfection, cells were selected using puromycin (1 µg/mL) or neomycin (0.7–1 mg/mL) to generate stable *HR*-expressing or *CELF2*-expressing cell lines and respective control cell lines for subsequent in vitro and xenograft experiments.

### 2.2. Clinical Sample Information and Characteristics

DNA, RNA, and primary breast tumor tissues or normal breast tissues were collected and stored in the Molecular Pathology Shared Resource at Columbia University Irving Medical Center (CUIMC). Samples from patients with clinical and tumor registry data in the CUIMC Database Shared Resource were selected and included in this study. DNA samples from at least 20 luminal A, luminal B, HER2-enriched, or triple-negative breast cancer (TBNC) subtypes were retrieved and used in the targeted sequencing analysis. Tumor subtypes were defined by classical immunohistochemistry markers (ER, PR and HER2) together with Ki67 index and clinicopathologic variables (tumor size, grade, stage, and nodal involvement) [28,29]. Median patient age at the time of diagnosis was 51 years, with a range of 29–92 years. Over 90% of the patients had ductal carcinoma whereas others had carcinoma of another histological type, such as lobular carcinoma, medullary carcinoma, or mixed ductal and lobular carcinoma. Median breast tumor tissue purity was 75%, with a range of 50–100%. Primary BC tissue sections with matched normal breast tissue sections were obtained through the CUIMC Tissue Bank for immunofluorescence (IF) staining. The specimens used in this study and related data were de-identified and do not represent human subject research as per the IRB Tissue Banking protocol (AAAB2667).

### 2.3. Targeted Sequencing and CN Analysis

Genomic DNA from BC tissues and cell lines was used for *HR* mutation analysis via targeted sequencing using custom probes from Agilent to capture the DNA fragments of *HR* and 10 other BC-related genes (Table 1). Captured DNA was used for library construction and sequencing following the manufacturer instructions. Similarly, genomic DNA from BC tissues and cell lines was subjected to *HR* CN analysis by ddPCR assays using a predesigned HR CN assay (dHsaCNS987944070, Bio-Rad, Hercules, CA, USA). *RPP30* was included as an internal CN reference gene. RNA samples from selected HR-deficient BC tissues were used in RNA-seq studies as previously described [30]. 

### 2.4. HR Reconstitution in HR-Deficient BC Cells

MDA-MB-231 and T47D are two HR-deficient BC cell lines with *HR* CN loss and no *HR* gene expression. MDA-MB-231 and T47D cells were transduced with a pLenti6. 3/V5™-based *HR*-expressing lentivirus or with vector control lentivirus, and puromycin was used for selection (Vector Builder). Puromycin-resistant HR-expressing MDA-MB-231 and T47D cells and the respective vector-transduced control cells were subjected to cell proliferation analysis, H3K9 methylation analysis, as well as qRT-PCR and Western blotting.

### 2.5. Cell Proliferation and Wound-Healing Assays

Cell proliferation was assessed using the IncuCyte^®^ live cell analysis system (Sartorius, Goettingen Germany) as described in Supplementary Materials and Methods [31]. For wound-healing experiments, cells were seeded in triplicates in 96-well plates and grown to confluence. Wounds were then created using the IncuCyte^®^ Wound Maker. Thereafter, cells were washed with PBS to remove cellular debris. Fresh media with UNC0642 (5 μM and 10 μM, Cayman Chemicals, Ann Arbor, MI, USA) was added. The plate was incubated, and wound healing was recorded every 12 h in the IncuCyte ZOOM time-lapse imaging system for a total of seven days. The wound healing rate was expressed as the width of wounds at specific time points. Statistical significance was determined using Graphpad Prism 8.0.0 (GraphPad Software, San Diego, CA, USA).

### 2.6. Clonogenic Assay

To assess the colony-forming ability of BC cells following UNC0642 treatment, cells were seeded in six-well plates and treated with UNC0642 (5 and 10 μM) or DMSO (as control) for 48 h. Treated and control cells were split and re-seeded at 1000 cells/well in a new six-well plate with UNC0642 or DMSO for another 48 h. Cells were then incubated in fresh medium without UNC0642 or DMSO for six additional days before being fixed in 4% formaldehyde and stained with 0.5% crystal violet. The colony number was counted using ImageJ software (version 1.53h 04).

### 2.7. Mouse Xenograft Experiments

NSG mice (NOD SCID IL2Rg^null^) were purchased from The Jackson Laboratory (Stock #005557). Animal care and use were conducted in accordance with NIH guidelines, and the mice were housed in an Association for Assessment and Accreditation of Laboratory Animal Care-accredited facility. All animal experiments were performed in strict accordance with protocols approved by the Institutional Animal Care and Use Committee of the University of Minnesota (2111-39576A). Mouse xenograft experiments were performed as previously described [32]. Briefly, MDA-MB-231 and T47D cells with reconstituted with *HR* or *CELF2* expression and their respective controls were orthotopically injected into the mammary fat pad of NOD SCID mice. For each injection, 1 × 10^6^ cells were resuspended in 100 µL of a serum-free media and Matrigel mixture (1:1 ratio). For UNC0642 treatment, mice bearing MDA-MB-231 cells were administered either UNC0642 (5 mg/kg body weight, in 0.2% DMSO) or 0.2% DMSO via i.p. injection three times a week for a total of 4 weeks. Tumor growth was monitored and recorded weekly for up to eight weeks or until the tumor reached 2 cm in diameter, after which the animals were euthanized for tumor collection and analysis.

### 2.8. RNA Extraction, qRT-PCR, and RNA-Seq Analysis

Total RNA was isolated from cultured BC cells using the RNeasy kit (Qiagen, Hilden, Germany). For qRT-PCR, 2 µg of RNA was reverse-transcribed into cDNA. qRT-PCR was performed using RT^2^ SYBR Green qPCR Mastermix (Qiagen), and the results were analyzed using the Bio-Rad CFX 96 Touch system (Bio-Rad). RNA-seq analysis was performed as described in Supplementary Materials and Methods [30]. Differentially expressed genes (DEGs) between HR-expressing and control cells or between primary breast tumors and adjacent normal mammary tissues were determined using the DESeq2 software package (version 3.10) [33], with a fold change (FC) cutoff set at >2 or <0.5.

### 2.9. Western Blotting and Immunofluorescence Staining

Primary antibodies were purchased from Abcam (Cambridge, MA, USA; HR, ab202840), Boster (Pleasanton, CA, USA; Hr, A04817), Aviva (Aviva Systems Biology, San Diego, CA, USA; CELF2, ARP40323), Sigma (St. Louis, MO, USA; FLAG, F1804; β-actin, A1978), and ABClonal (Woburn, MA, USA; H3K9me1, A2355; H3K9me2, A2359; CDK15, A13163; NR2F1, A16437; CA9, A1658). For Western blotting, approximately 30 µg of total cell lysate from each sample was loaded. IF staining was performed as described in Supplementary Materials and Methods [19]. IF images were acquired using a fluorescence confocal microscope (Zeiss, Thornwood, NY, USA). All IF experiments were performed in at least three independent experiments.

### 2.10. Variant Calling and Annotation, Bioinformatics, and Statistical Analysis

Variant calling and annotation were performed as described in detail in Supplementary Materials and Methods. Variants were annotated with ANNOVAR [34] to add ExAC (exac03) and Gnomad Exome (gnomad211_exome) population frequencies and ClinVar clinical significance (clinvar_20200316), and were filtered to remove variants with an overall population frequency >1% in either ExAC or Gnomad, or a clinical significance of “Benign” or “Likely Benign” in ClinVar. Two samples (986 and 1256) that had median coverage <20× were excluded from further analysis. Variants for the remaining samples were compiled using a custom R script (R v. 4.0.0) to filter variants. Multiple criteria were applied to filter variants, i.e., only variants with >5% variant allele frequency with at least three alternative reads supporting each variant were retained. To remove technical artifacts as well as common germline mutations, the Mutect2 Panel of Normals variants were used to filter the calls. Figures were generated using the R packages maftools (v.2.4.12) [35] and ggplot2 (v.3.3.3) [36]. For statistical analysis, a proportion Z-test was performed to determine whether the proportion of BC tissues with CN loss was the same between different BC subtypes. Fisher’s exact test was used to assess whether there was a significant association between CN loss and tumor recurrence or remission. To explore the co-occurrence of mutations within the same samples, we performed Fisher’s exact test to determine whether there was a significant overlap between sample lists with specific mutations. *p*-Values from the tests were FDR corrected to control for false positives. To identify whether there are significant associations between genetic variants and time to tumor recurrence and survival in cancer patients, survival analysis was performed, and the log-rank test was used to compare Kaplan–Meier curves between individual mutations as well as co-mutations. 

## 3. Results

### 3.1. Frequent HR Mutations in BC Tissues

HR exhibits antitumor activity during skin tumorigenesis [14,21], but its tumor suppressor function remains to be defined in other settings. Analysis of BC genomic datasets revealed several recurrent HR mutations such as G337D, S366C, E448G, R747H, R927C, P1046L, and G368fs (frame-shift insertion) [14,27]. To better define *HR* mutation prevalence and spectrum in BC, we performed targeted next-generation sequencing using genomic DNA samples from primary breast tumor tissues. In addition to *HR*, other established BC susceptibility genes such as *BRCA1*, *BRCA2*, *PTEN*, *ESR1,* and *TP53*, were also included in the targeted sequencing panel in order to compare the *HR* mutation rate relative to that of these genes in the same patient cohort (Supplementary Table S1). We used the Agilent XTHS2 hybrid capture-based target enrichment method to prepare the sequencing libraries. Libraries from 18 luminal A, 21 luminal B, 25 HER2-enriched, 19 TNBC, and six BC cell line samples passed sequencing library quality control and proceeded to next-generation sequencing.

**Figure 1:**
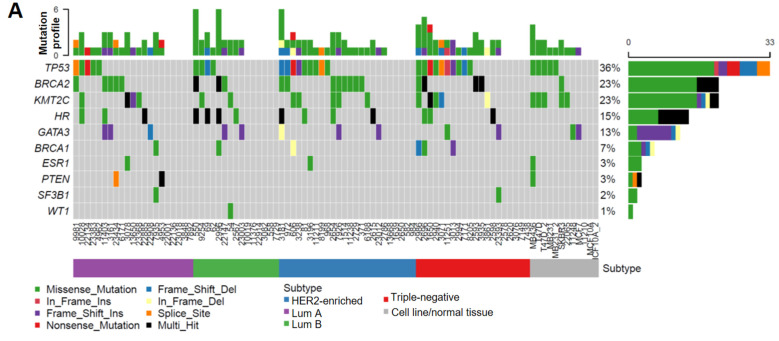

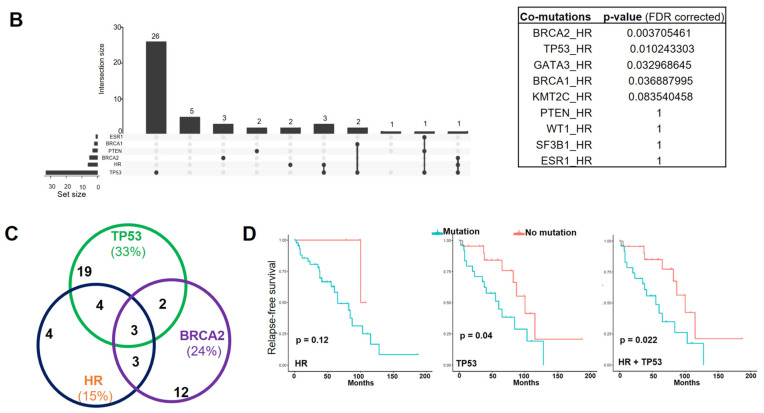
*HR* gene mutations are correlated with an increased recurrence rate in human BC. (**A**) Oncoplot showing mutations identified via targeted sequencing in the coding regions of *HR* and other key genes known to contribute to BC pathogenesis in 85 BC samples and 6 BC cell lines. X-axis represents each individual patient. Y-axis depicts the number of variants identified in all 11 genes in each patient. (**B**) An UpSet plot showing the co-occurrence of mutations among selected genes within each sample. Intersection size describes the number of samples that have at least one mutation in each of the genes with a dot below them. Multiple Fisher exact tests were performed to determine the significance of each pair of the co-occurring mutations among the samples. Based on the FDR-corrected *p*-values, *HR* and *BRCA2* had a significant co-mutation rate among samples. (**C**) Venn diagram showing the co-mutation rate of *HR*, *TP53*, and *BRCA2* among samples. (**D**) Tumor recurrence and patient survival analysis based on *HR* and *TP53* mutations or co-mutations.

As summarized in Figure 1A, the *TP53* somatic mutation rate (36%) and its increased frequency in HER2-enriched/TNBC subtypes (Table 1) were consistent with previous findings [37,38,39,40], which validated the accuracy and reliability of our targeted sequencing results. Interestingly, we found that the mutation frequency in *HR* coding regions (15%) is lower than that for *BRCA2* (23%), but higher than those of other BC susceptibility genes including *BRCA1* (7%), *ESR1* (3%), and *PTEN* (3%) (Figure 1A). Unlike higher *TP53* mutations in HER2-enriched/TNBC subtypes, *HR* mutations had a relatively even distribution among different subtypes (Table 1). Notably, multiple *HR*-mutation types were detected in the same patient in approximately 50% patients with *HR* mutations (indicated by black bars in Figure 1A), whereas the other genes were affected predominantly by missense mutations (indicated by green bars in Figure 1A), suggesting a higher genetic instability of the *HR* gene locus in a subset of patients.

Additionally, we found that *HR* mutations co-occurred with mutations in *TP53* and *BRCA2* in a subset of specimens (Figure 1B,C), although the clinical significance of these co-occurring mutations is not clear. Survival analysis revealed no significant association between *HR* mutations and patient survival. However, we found that patients with *HR* and *TP53* co-mutations had a significantly increased risk of tumor recurrence (Figure 1D), suggesting a possible collaborative function between the loss of HR and p53 in promoting breast tumorigenesis. In contrast to the higher rate of *TP53* mutation in HER2 and TBNC subtypes, *HR* and *TP53* co-mutations distributed evenly among different BC subtypes (Figure 1A). Detailed mutation profiling of the *HR* locus (including non-coding regions) in each tumor sample (Supplementary Figure S1A) revealed that some tumors harbored more mutations than others. Furthermore, we found three mutations in the HR JmjC domain and several recurrent missense *HR* mutations that are present in other cancer types, including P63R, G337D, and G823S (Supplementary Figure S1B). The impact of these recurrent mutations on HR function remains to be determined in future functional studies.

### 3.2. HR Copy Number (CN) Loss Is Associated with an Increased Risk of Tumor Recurrence

**Figure 2:**
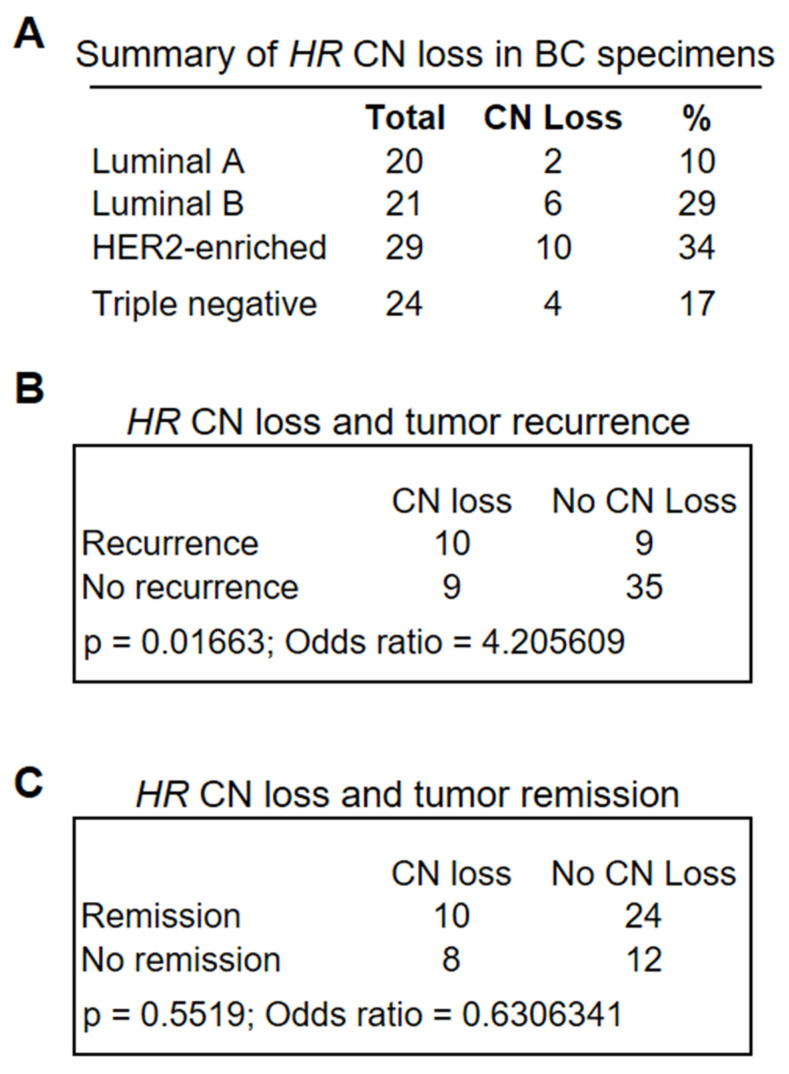
(**A**) Frequency of *HR* CN loss in BC specimens as determined via ddPCR analysis of 94 BC specimens encompassing different BC subtypes. Lum A group had a lower-than-average CN loss (23.4%) across all subtypes (*p* = 0.0784 based on the proportion Z-test), whereas HER2-enriched subtypes had the greatest average CN loss (*p* = 0.0794). (**B**,**C**) Fisher’s exact tests showed a significant association between *HR* CN loss and tumor recurrence (*p* = 0.01663 (**B**)), but not tumor remission (**C**).

In addition to missense mutations, analysis of the Cancer Cell Line Encyclopedia (CCLE) mutation database identified *HR* gene CN loss among human BC cell lines [41,42], highlighting a potential role for HR deficiency in BC. To determine the frequency of *HR* CN changes in primary tumor tissues, we performed droplet digital PCR (ddPCR) assays to assess *HR* CN status in different BC subtypes (n = 94). The ddPCR results summarized in Figure 2A demonstrated *HR* CN loss in all four subtypes, with a relative low frequency in the luminal A subtype and a high frequency in the HER2-enriched subtype, largely consistent with the observation of *HR* CN loss in human BC cell lines. Among the BC cell lines, we found *HR* CN loss in T47D and MDA-MB-231, but not in MCF10A and MCF7 cells. 

To determine whether *HR* CN loss was associated with clinical outcomes, we retrieved patient clinical information from the CUIMC tumor registry, including survival status, tumor recurrence, and remission. We conducted Fisher’s exact test to determine the significance of differences in the association between *HR* CN loss and survival, tumor recurrence, or remission with that from *HR*-wild-type tumors. As shown in Figure 2B,C, *HR* CN loss was significantly associated with tumor recurrence (Figure 2B, *p* = 0.01663, n = 63), but no significant association with tumor remission was observed (Figure 2C, *p* = 0.5519, n = 54). Similarly, *HR* CN loss was associated with reduced patient survival, albeit not statistically significant. Taken together, these analyses suggested that *HR* deficiency may serve as a novel biomarker for an increased risk of tumor recurrence.

### 3.3. HR Re-Expression Reduced Histone H3K9 Methylation in BC Cells as Well as Tumor Growth In Vivo

**Figure 3:**
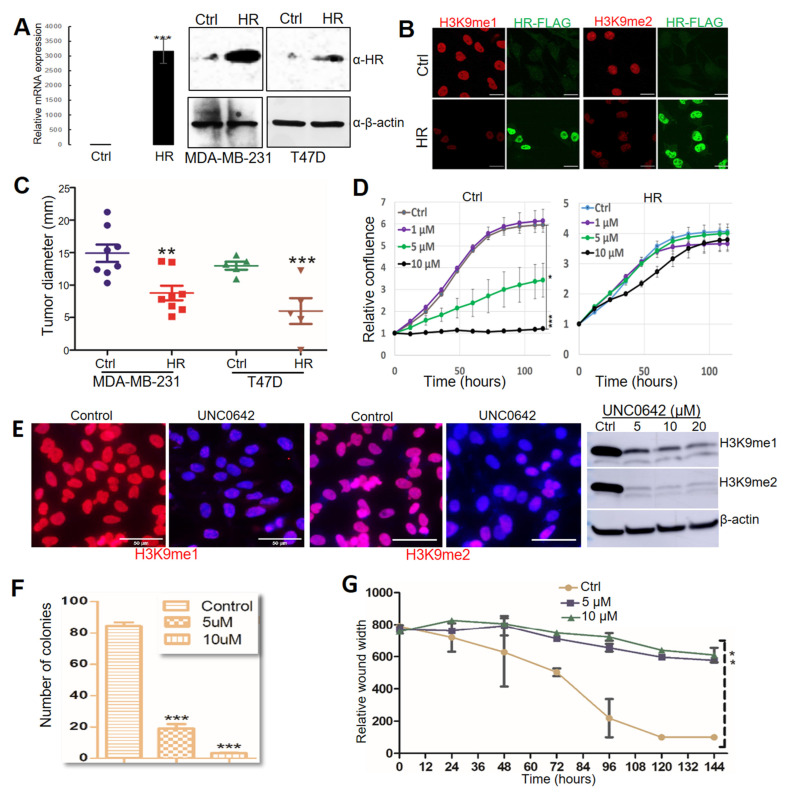
HR regulates breast tumor progression through H3K9 methylation. (**A**) HR mRNA and protein expression in MDA-MB-231 and T47D cells determined by real-time PCR and Western blot (File S1). (**B**) Relative levels of mono-methylated H3K9 (H3K9me1) and di-methylated (H3K9me2) in MDA-MB-231 cells. MDA-MB-231 cells were transduced with control lentivirus (Ctrl) or lentivirus-HR (HR), followed by immunofluorescence staining with anti-H3K9me1 (red), Flag (green), and counter staining with DAPI (blue). Scale bar: 20 μm. (**C**) Tumor growth after MDA-MB-231 and T47D cells transduced with control lentivirus (Ctrl) and lentivirus-HR (HR) were inoculated into the fat pads in NOD SCID mice. (**D**) IncuCyte ZOOM time-lapse imaging analysis for MDA-MB-231 cell proliferation after treatment with DMSO (Ctrl) or UNC0642 (1, 5, or 10 μM). Data are presented as the mean ± standard deviation (n = 3). (**E**) Detection of changes in H3K9me1 and H3K9me2 in MDA-MB-231 cells after treatment with DMSO (control) or UNC0642 by IF and Western blotting (File S1). For IF experiments, cells were stained with anti-H3K9me1 or anti-H3K9me2 (red) and counter stained with DAPI (blue). Scale bar: 50 μm. (**F**) Colonies formed by MDA-MB-231 cells after treatment with DMSO (control) or UNC0642 (5 or 10 μM). (**G**) Wound healing of the MDA-MB-231 cell monolayer after treatment with DMSO (Ctrl) or UNC0642 (5 or 10 μM). *: *p* < 0.05. **: *p* < 0.01 vs control. ***: *p* < 0.001 vs control.

Whereas HR mutation and CN loss were detected in clinical specimens, only HR CN loss was detected in two of the BC cell lines (MDA-MB-231 and T47D). HR expression in MDA-MB-231 and T47D cells was undetectable via qRT-PCR or Western blotting (Figure 3A). To test if HR reconstitution in these HR-deficient BC cells could suppress H3K9 methylation, we utilized a lentivirus-based expression system to restore HR expression in T47D and MDA-MB-231 cells. HR expression was confirmed by qRT-PCR and Western blotting in puromycin-resistant HR-expressing (HR) lentivirus-transduced cells but not in control lentivirus-transduced (Ctrl) cells (Figure 3A). Consistent with HR’s H3K9 demethylase activity, HR re-expression led to a drastic loss of histone H3K9 methylation, as shown by IF staining (Figure 3B). In vitro cell proliferation assays revealed no significant changes between HR-expressing and control MDA-MB-231 and T47D BC cells. 

To test if HR restoration reduces tumor growth by these HR-deficient cells in vivo, we injected control and HR-expressing cells orthotopically into mammary fat pads in NOD/SCID mice. Approximately 8 weeks after injection, mice were euthanized for necropsy to examine tumor growth at the injected mammary pads and metastasis to other organs. As shown in Figure 3C, HR re-expression substantially suppressed both MDA-MB-231 and T47D tumor growth. Necropsy analysis found tumor metastasis to the skin and lung in mice injected with control MDA-MB-231 cells but not HR-expressing cells, whereas no metastatic lesions were found in mice injected with either control or HR-expressing T47D cells. These xenograft experiments suggested that HR effectively inhibited the in vivo tumor growth and metastasis of MDA-MB-231 cells. 

UNC0642 is a potent and selective small-molecule inhibitor of H3K9 methylation that targets the H3K9 methyltransferases G9a and GLP [43]. Based on the H3K9 demethylase activity of HR, we postulated that inhibition of H3K9 methylation via UNC0642 can suppress the growth and viability of HR-deficient cancer cells. To test this, we treated MDA-MB-231 cells with UNC0642 and measured their proliferation over a period of seven days. As shown in Figure 3D, UNC0642 at both 5 and 10 μM significantly inhibited MDA-MB-231 cell growth (Figure 3D, left panel). Intriguingly, HR re-expression in MDA-MB-231 cells conferred resistance to UNC0642 treatment (Figure 3D, right panel), probably because these cells may have adapted to low genomic H3K9 methylation following HR reconstitution. UNC0642-induced inhibition of H3K9 methylation was confirmed by IF staining and Western blotting (Figure 3E). In addition to growth inhibition, in vitro clonogenic assays and wound-healing assays demonstrated that UNC0642 significantly reduced the clonogenicity and migration of MDA-MB-231 cells (Figure 3F,G, and Supplementary Figure S2A,B). We also demonstrated that UNC0642 significantly inhibited MDA-MD-231 xenograft tumor growth in NOD SCID mice, which was coupled with marked losses of H3K9me1/m2 in comparison with DMSO-treated mice (Supplementary Figure S2C), highlighting the in vivo antitumor efficacy of UNC0642 and its potential as an epigenetic anticancer drug. 

### 3.4. Identification of Target Genes Mediating the Antitumor Function of HR in MDA-MB-231 BC Cells

**Figure 4:**
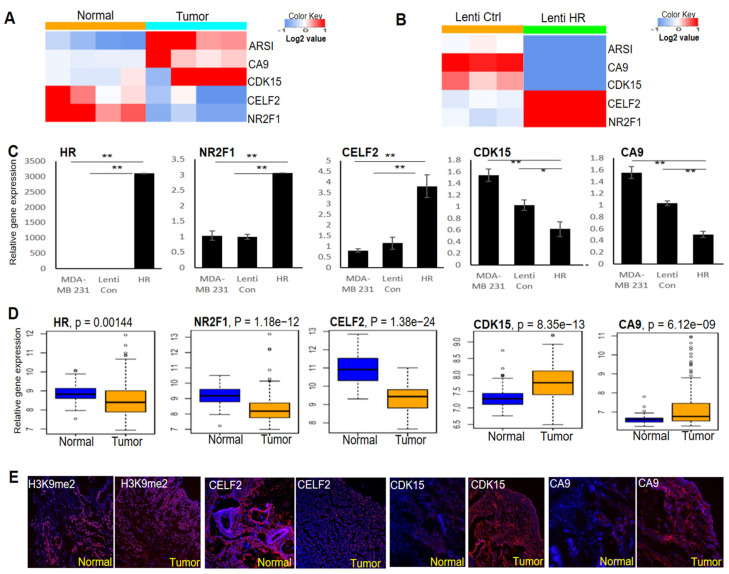
Identification of HR-regulated genes in BC cells and breast tumors. (**A**) RNA-seq analysis identified multiple genes whose expression was consistently increased or decreased in breast tumors compared with matched normal tissues (n = 4). (**B**) RNA-seq analysis showing that the expression of the genes identified in **A** was reversed via *HR* re-expression in human MDA-MB-231 cells (n = 3). (**C**) qRT-PCR validation of HR-regulated genes in HR-expressing and control MDA-MB-231 cells. **: *p* < 0.01 vs control; *: *p* < 0.05 vs control. (**D**) Comparative analysis of *HR* and target gene mRNA expression in human BC tissues compared with matched normal breast tissues from the TCGA dataset (n = 112 pairs). (**E**) Representative IF images showing reduced CELF2 expression, increased H3K9me2 methylation as well as increased CDK15 and CA9 expression in HR-deficient BC tissue compared with matched normal breast tissue.

To identify HR-regulated genes in BC cells, we performed RNA-seq to determine differential gene expression between HR-expressing and control MDA-MB-231 cells. Differentially expressed genes between four pairs of *HR*-deleted breast tumors and matched normal breast tissues were also identified via RNA-seq. Among the top-ranked differentially expressed genes between these two RNA-seq datasets, *CELF2* and *NR2F1* were consistently decreased in *HR*-deleted breast tumors (Figure 4A), but upregulated in HR-expressing MDA-MB-231 cells (Figure 4B). In contrast, the expression of *CDK15*, *CA9*, and *ARSI* was elevated in tumors but repressed in HR-expressing MDA-MB-231 cells (Figure 4A,B). As shown in Figure 4C, we validated the differential expression of *CELF2*, *NR2F1*, *CDK15*, and *CA9* between HR-expressing and control MDA-MB-231 cells by qRT-PCR. 

To assess the expression status of HR and its target genes in breast tumors, we compared TCGA RNA-seq data from breast tumors and matched normal mammary tissues (n = 112 pairs) by paired gene expression analysis. We found that *HR* expression was significantly down-regulated in breast tumors, in parallel to significant changes in the expression of the above HR-regulated genes (Figure 4D). IF staining in matched pairs of *HR*-deleted breast tumors and matched normal tissue revealed an increase in H3K9 methylation in the tumor, coupled with increased expression of CDK15 and CA9 but decreased expression of CELF2 (Figure 4E), consistent with the qRT-PCR and RNA-seq results. These HR-regulated genes may contribute to BC pathogenesis and could also serve as novel biomarkers to identify patients who may respond to BC treatment via H3K9 methylation inhibitors.

### 3.5. CELF2 Exhibits Potent Antitumor Activity in MDA-MB-231 Cells

**Figure 5:**
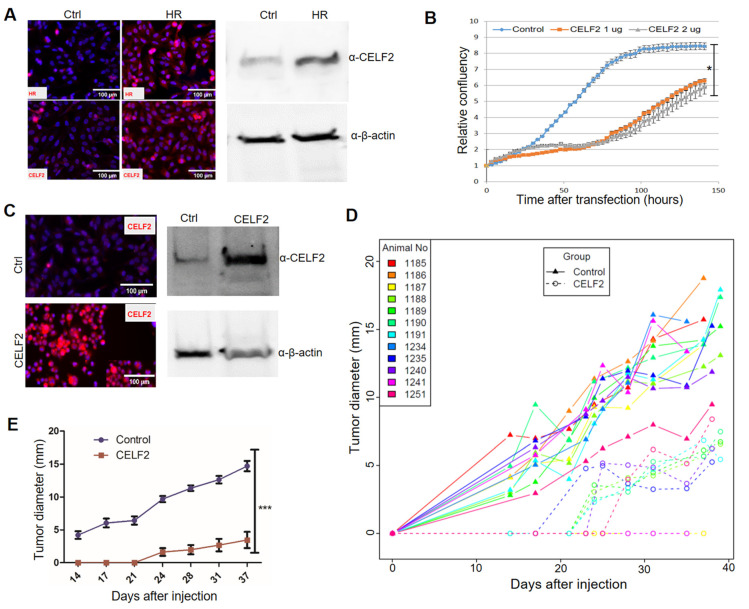
CELF2 suppresses BC cell proliferation and tumor growth. (**A**) Upregulation of CELF2 protein expression in HR-reconstituted cells. CELF2 protein expression in HR-reconstituted (HR) MDA-MB-231 cells was examined via IF (left) or Western blotting (File S1) (right) using a CELF2 antibody (scale bar: 50 μm). β-Actin was used as a loading control. (**B**) Proliferation of MDA-MB-231 cells transfected with control or CELF2-expressing plasmids (1 and 2 μg). Cell proliferation was expressed as the relative confluency by using the Incucyte system. *: *p* < 0.05 vs the control. (**C**) Confirmation of CELF2 overexpression in a selected CELF2-transfected MDA-MB-231 cell line via IF (left) or Western blotting (File S1) (right) using the CELF2 antibody. (**D**,**E**) In vivo tumor growth curves of control or CELF2-overexpressing (CELF2) MDA-MB-231 cells following injection into SCID mouse fat pads. Tumor sizes measured from each mouse are illustrated with a solid (control) or dotted (CELF2 overexpression) line in the scatter plot (**D**). Average tumor sizes from control or CELF2 overexpression groups are illustrated in (**E**). ***: *p* < 0.001 vs control.

Among the newly identified HR target genes, *CELF2* encodes an RNA-binding protein. CELF2 has been shown to suppress lung cancer cell proliferation by repressing AKT phosphorylation in a PTEN-dependent manner [44]. CELF2 activity is known to be epigenetically regulated via DNA methylation [44,45]. Notably, loss of HR expression in human BC specimens is coupled with CELF2 downregulation (Figure 4C). Consistent with the positive correlation between HR and CELF2 expression, HR reconstitution led to increased CELF2 expression in MDA-MB-231 cells (Figure 5A). Additionally, we showed that transient CELF2 expression inhibited MDA-MB-231 cell proliferation dose dependently (Figure 5B). To further explore the antitumor activity of CELF2 in BC cells, we generated stable CELF2-expressing MDA-MB-231 cells. Compared with vector-transfected control MDA-MB-231 cells, stable CELF2 expression significantly suppressed MDA-MB-231 cell proliferation and migration (Supplemental Figure S2D). Following injection into the mammary fat pads in NOD SCID mice, tumor growth by CELF2-expressing MDA-MB-231 cells was markedly reduced compared with the control MDA-MB-231 cells (Figure 5C,D). Taken together, these experiments provide compelling evidence that CELF2 is upregulated by HR and exerts a strong tumor-suppressive effect on BC cells.

## 4. Discussion

The human *HR* gene is located on chromosome 8p, a region that is frequently lost in breast and other cancer types [46]. Analysis of genes near the *HR* locus on 8p reveals no other known tumor suppressor genes except for *HR* (unpublished observations), suggesting that HR loss might contribute to breast tumorigenesis. Few genetic studies of BC in the past have reported any significant HR mutations in BC patients. In this study, we performed targeted, next-generation sequencing to define the HR mutation frequency and profile in a cohort of BC samples collected between 2001–2013. The mutation rates of the established BC susceptibility genes analyzed in this study are largely consistent with those reported previously. Interestingly, we found *HR* mutations in approximately 15% of the cohort, which is lower than that for *TP53* and *BRCA2* but higher than that for *BRCA1*, *PTEN,* and *ESR1* (Figure 1A). After additional mutation filtering to remove variants labeled as “benign” or “likely benign” in the ClinVar database, potential pathogenic *HR* mutations remain prevalent compared with *BRCA2/PTEN/BRCA1* mutations (Supplementary Figure S1C), suggesting that *HR* mutation might function as a risk factor for BC.

Our targeted sequencing results confirmed frequent *TP53* mutations in BC with subtype-dependent variations (higher in HER2-enriched and TNBC tumors than in luminal A and B tumors) (Figure 1A). *TP53* mutation is also detected in MDA-MB-231, MDA-MB-468 and T47D BC cell lines, consistent with previous findings [47,48]. Unlike *TP53*, *HR* mutation does not show any subtype-dependent variations and is not detected in the BC cell lines examined in this study. Intriguingly, approximately 50% of HR-mutant tumors harbor two or more types of mutations in HR, indicating a possible genetic instability at the HR locus in these patients. Survival analysis revealed no significant association between *HR* mutation and patient survival in this relatively small patient cohort, but *HR* and *TP53* co-mutations are associated with a significant risk of tumor recurrence (Figure 1D). The clinical significance of *HR* mutations and their collaborative function with *TP53* mutations await further exploration in large patient cohorts. 

In support of the antitumor function of HR in BC development, we demonstrated that HR significantly inhibited xenograft tumor growth and metastasis by using HR-deficient MDA-MB-231 cells in NOD SCID mice (Figure 3). While the mechanism awaits further investigation, the new HR-regulated genes identified in this study might contribute to HR tumor suppressive activities in BC. *CDK15* encodes a cyclin-dependent kinase that exhibits anti-apoptosis as well as tumorigenic activity. CDK15 confers resistance to TRAIL-induced apoptosis [49], but inhibition of CDK15 paradoxically enhances BC cell invasion and metastasis [50]. CELF2 is an RNA-binding protein that regulates its target gene activity post transcriptionally through alternative splicing. *CELF2* encodes a tumor suppressor and is epigenetically silenced by DNA methylation in human cancers [45]. CELF2 has been shown to suppress lung cancer cell proliferation by repressing AKT phosphorylation in a PTEN-dependent manner [44]. Using the MDA-MB-231 cell culture and xenograft models, we demonstrated that CELF2 expression exhibits potent antitumor activity in MDA-MB-231 cells. In addition to the newly identified HR target genes, HR may regulate cell cycle progression, proliferation, migration, and survival through other target genes, including *IL1R2*, *COL6A1*, *CSNK2A2*, *DLEC1*, *FADD*, *FGF13*, and *PVT1*, which were identified by ChIP-seq studies [19]. Mutational inactivation of HR leads to aberrant NF-κB activation [21], and could in turn promote epithelial–mesenchymal transition to drive BC metastasis [51]. HR might also regulate BC metastasis through Wnt signaling based on previous studies showing that HR regulates WISE, a modulator of the Wnt signaling pathway [52]. Furthermore, recent studies show that HR and p53 share common target genes (*PUMA*, *GADD45A*, and *CDKN1A*) and downstream effectors (*BIRC5* and *STMN1*) [53,54], suggesting a possible crosstalk between HR and p53 in their tumor suppressor activities. 

In light of its demethylase activity, the tumor-suppressive function of HR might be mediated epigenetically through H3K9 methylation. HR demethylates H3K9me1 and H3K9me2 to activate or repress its target gene expression in a context-dependent manner [14,19,55,56]. Mutational inactivation of HR may lead to epigenetic activation of cancer-promoting genes or repression of tumor suppressor genes to promote BC development. Thus, HR-dependent alterations in H3K9 methylation offers novel opportunities to examine the mechanistic link between histone methylation and BC development. As a proof of concept, we demonstrated that HR-deficient MDA-MB-231 cells are highly sensitive to treatment with a specific H3K9 methylation inhibitor, UNC0642 (Figure 3). Notably, MDA-MB-231 cells with HR expression became resistant to UNC0642 treatment, suggesting that HR-dependent H3K9 demethylation rendered MDA-MB-231 cells insensitive to H3K9 methylation inhibition. These findings highlight the potential use of UNC0642 or other methylation inhibitors in epigenetic BC therapy. 

In mammalian cells, G9A is a major histone methyltransferase that catalyzes H3K9me1 and H3K9me2. Consistent with the opposing roles of HR (a histone demethylase) and G9A (a histone methyltransferase) in histone H3K9 methylation, *G9A* is suggested to function as an oncogene, in contrast to the antitumor function of HR. G9A is aberrantly upregulated in various human cancers and is associated with poor patient prognosis [57,58,59,60]. Thus, HR loss may contribute to tumorigenesis via increased H3K9 methylation. It is unknown, however, whether HR and G9A share common target genes in the genome. In addition to its role in H3K9 methylation, G9A can also methylate the p53 protein at Lys^373^ to inhibit p53 activity [59]. Whether HR–p53 interaction is mediated via the demethylase activity of HR remains to be determined. 

In conclusion, the present study demonstrated that HR exerts a tumor-suppressive effect on BC cells. This antitumor function is partly mediated via CELF2 upregulation. Ectopic expression of CELF2 alone is sufficient to significantly inhibit BC cell growth and migration. Furthermore, we demonstrated that pharmacological inhibition of H3K9 methylation effectively suppressed the growth and survival of *HR*-deficient BC cells. This study paves the way for future studies to explore specific inhibitors of the H3K9 methylation pathway as epigenetic therapeutics to abrogate the growth and progression of HR-deficient tumors.

## Figures and Tables

**Table 1:** Mutation frequency of the genes included in the targeted deep-sequencing panel among BC subtypes (based on receptor expression status) and 5 BC cell lines. Probes were designed to capture the exons of each of the genes for sequencing.

	Lum A	Lum B	HER2 Enriched	TNBC	Cell Lines
Total	21	16	24	20	5
HR	3	4	4	3	0
TP53	5	4	9	10	3
BRCA2	5	3	8	4	1
KMT2C	4	2	5	5	3
GATA3	3	2	3	2	1
BRCA1	1	1	1	3	0
ESR1	1	0	1	0	1
PTEN	2	0	0	0	1
SF3B1	1	0	0	1	0
WT1	0	1	0	0	0

## Data Availability

All pertinent data are available within the manuscript or upon request. The sequencing datasets generated and analyzed during the current study will be available in the NCBI Sequence Read Archive database (accession code PRJNA799398). All code used to analyze sequencing data as described above are from publicly available resources.

## Supplementary Materials

The following supporting information can be downloaded at: https://www.mdpi.com/article/10.3390/cancers14194648/s1, Table S1. Genes included in the targeted sequencing panel; Figure S1. HR mutation in breast cancers; Figure S2. Impact of UNC0642 treatment on colony formation, wound healing, and in vivo tumor growth. File S1: Original Western blots.
