# Supplementary material for: The Histone Demethylase HR Suppresses Breast Cancer Development through Enhanced CELF2 Tumor Suppressor Activity
Source: Cancers (Basel). 2022 Sep 24;14(19):4648. doi: 10.3390/cancers14194648 (PMC9564370; doi:10.3390/cancers14194648)
Supplement: Supplementary file 1 [file cancers-14-04648-s001.zip › supplementary Figures and Tables.pdf]

Table S1. Genes included in the targeted sequencing panel. *: Probes were designed to capture the entire HR gene locus, including both exons, introns, a 5′ upstream fragment (1 kb), and a 3′ downstream fragment (0.5 kb). For all other genes, probes were designed to capture the exons only.

Gene symbol	NCBI Gene ID	GenBank Accession
HR*	55806	NM_005144
TP53	7157	NM_001126118
WT1	7490	NM_006218
BRCA1	672	NM_007297
BRCA2	672	NM_000059
KMT2C (MLL3)	58508	NM_170606
GATA3	2625	NM_001002295
SF3B1	23451	NM_012433
ESR1	2099	NM_001122741

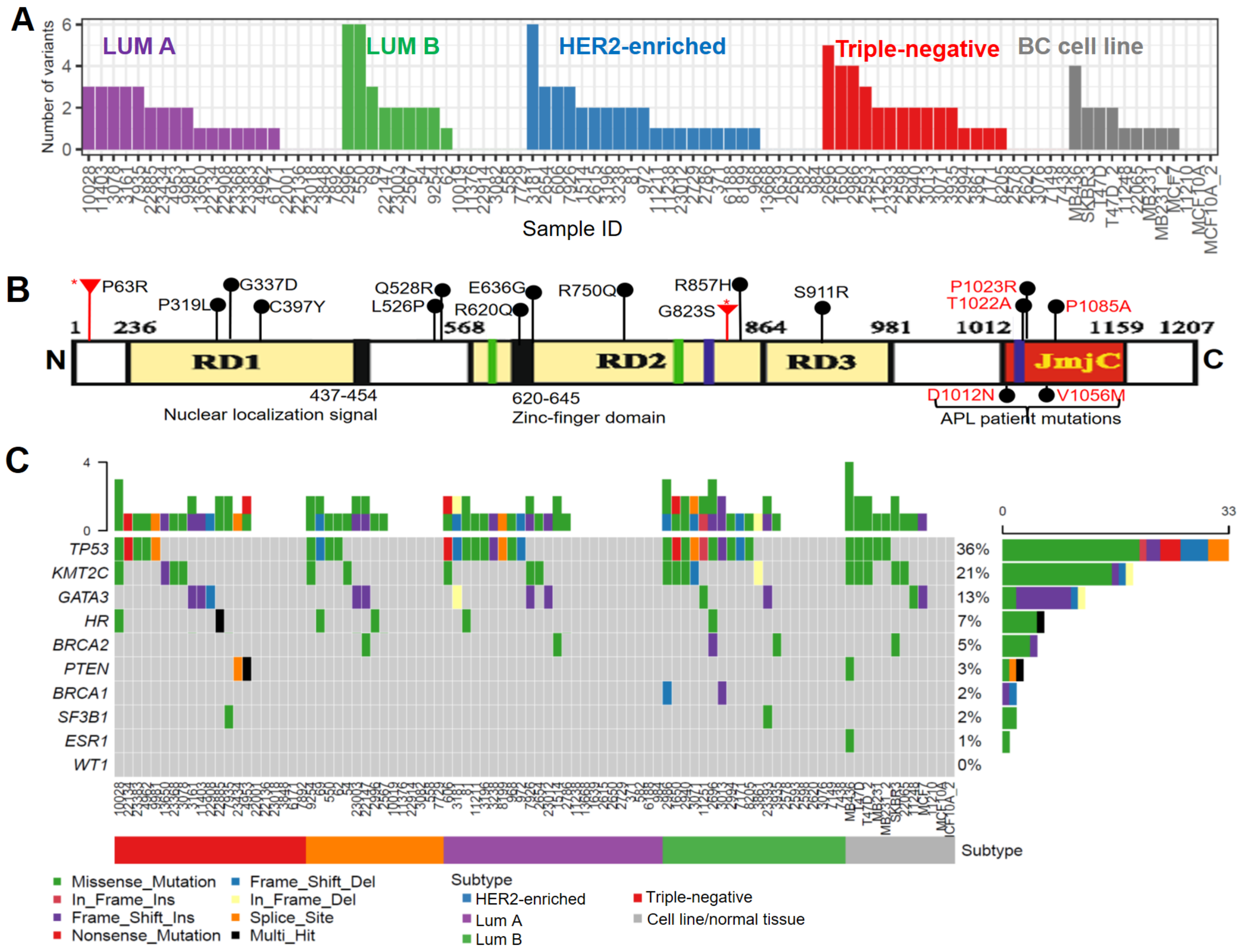

Supplementary Figure S1. HR mutation in breast cancers. (**A**) Mutation profiles of the entire HR gene locus in BC sample and selected BC cell lines. (**B**) Summary of HR missense mutations identified in BC specimens. *: **P63R** and **G823S** are two top recurrent mutations identified among the BC specimens. (**C**) Oncoplot showing mutations filtered by removing variants that are labeled as "benign/likely benign" in ClinVar database to highlight potentially consequential mutations.

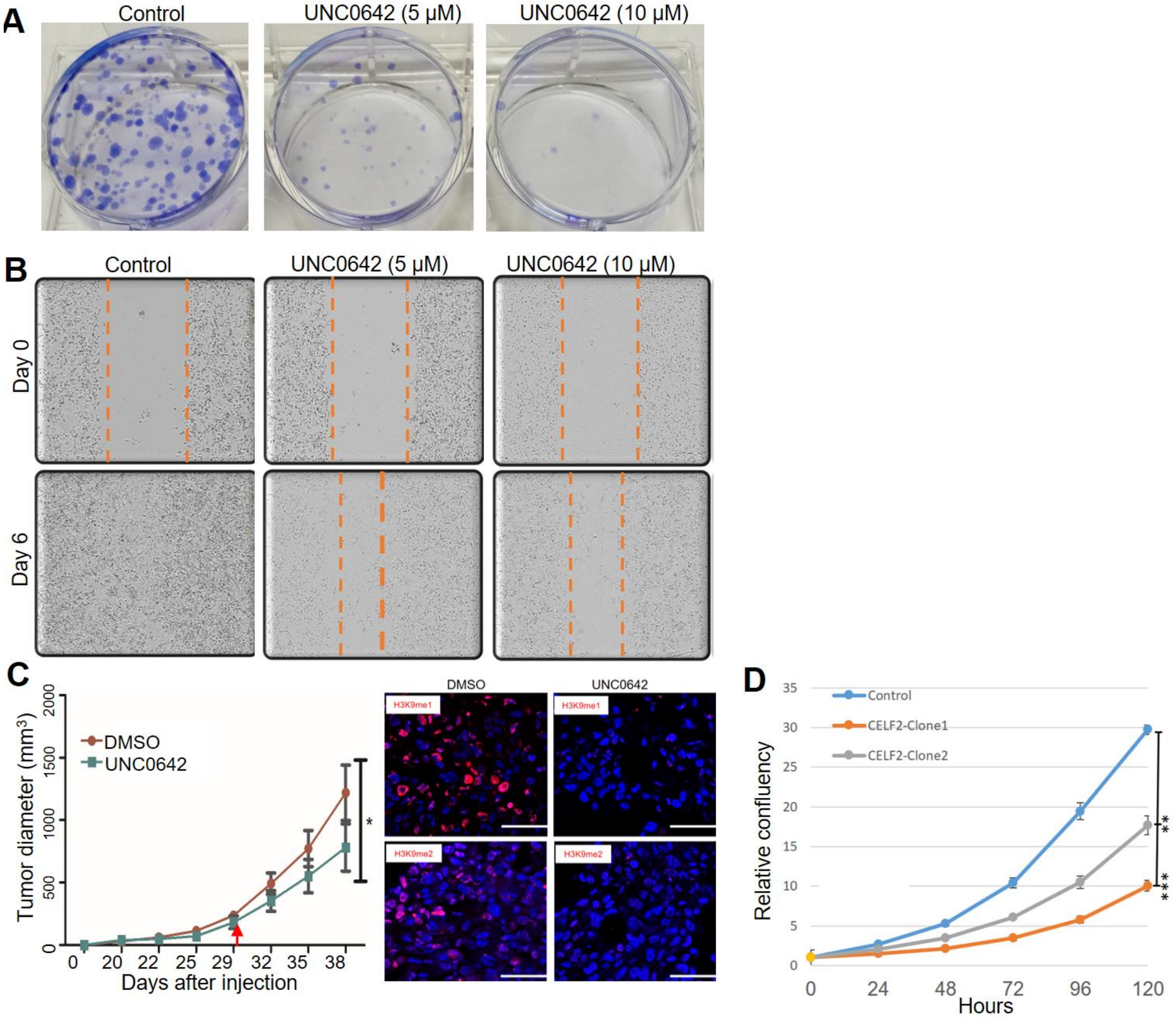

Supplementary Figure S2. Impact of UNC0642 treatment on colony formation, wound healing, and in vivo tumor growth. MDA-MB-231 cells were treated with DMSO (control) or UNC0642, followed by assays of colony formation or wound healing. **(A)** Colony formation of the cells stained with violet crystal. (**B**) Representative images from wound healing assays showing that UMN0642 inhibited cell migration and wound healing in vitro. Dashed outlines indicate wound widths at the beginning (0 h) and end of the experiments (144 h). (**C**) Inhibition of MDA-MB-231 tumor growth in xenograft mouse model by UNC0642 compared to DMSO (n=10). Last UNC0642 or DMSO treatment was on day 30 after tumor cell injection (indicated by the red arrow). IF staining detected the loss of H3K9me1/m2 in UNC0642-treated tumors (**D**) Proliferation of control and two MDA-MB-231 cell clones with ectopic CELF2 expression. *: p< 0.05; **: p< 0.01; ***: p< 0.001.
