# Supplementary figures and images for: The Histone Demethylase HR Suppresses Breast Cancer Development through Enhanced CELF2 Tumor Suppressor Activity
Source: Cancers (Basel). 2022 Sep 24;14(19):4648. doi: 10.3390/cancers14194648 (PMC9564370; doi:10.3390/cancers14194648)

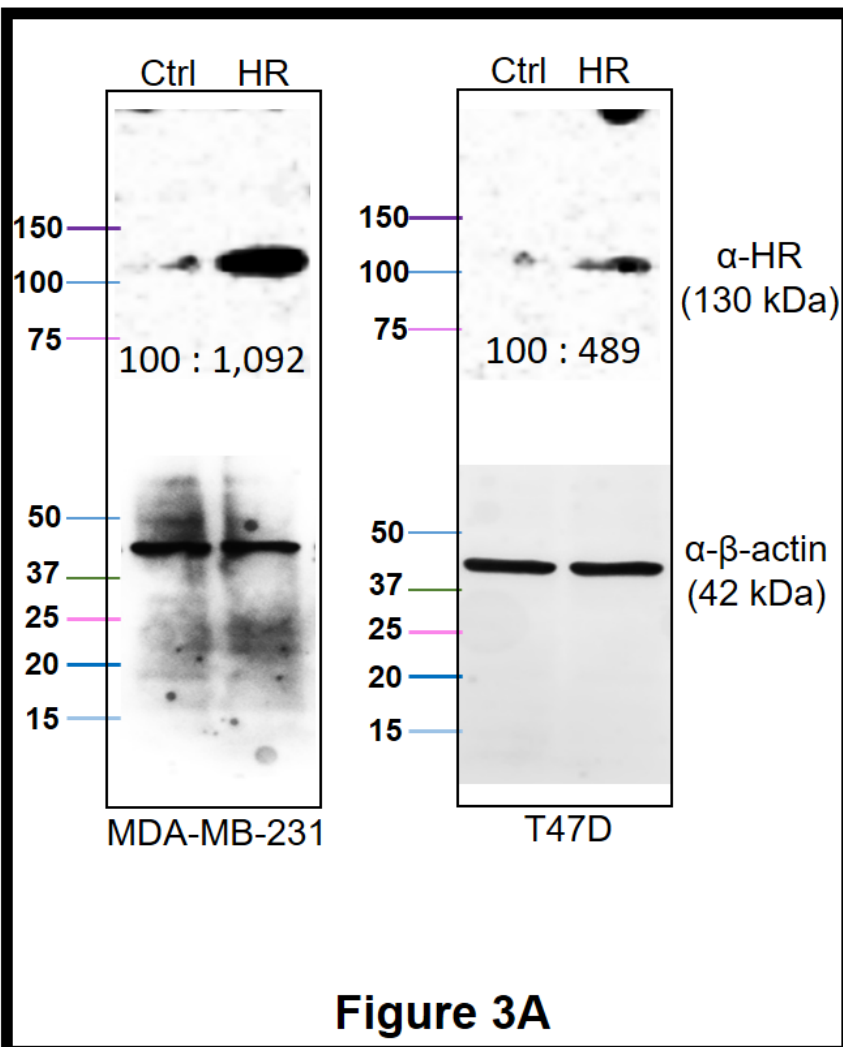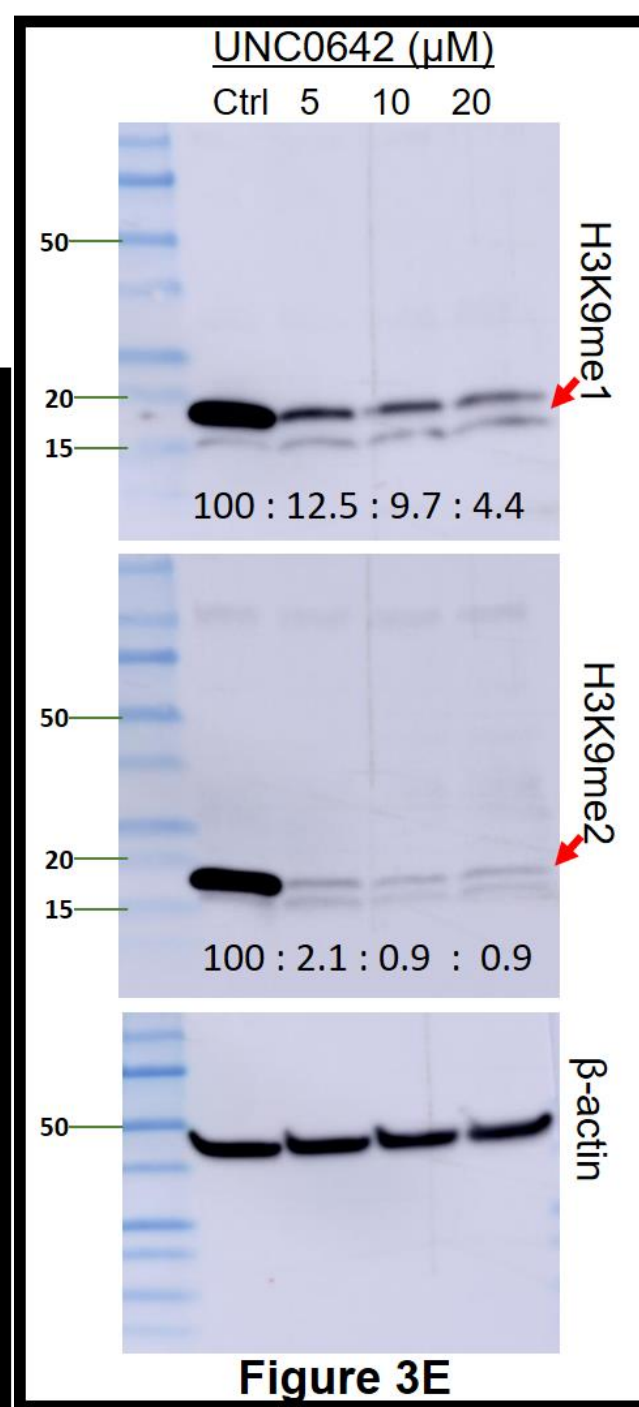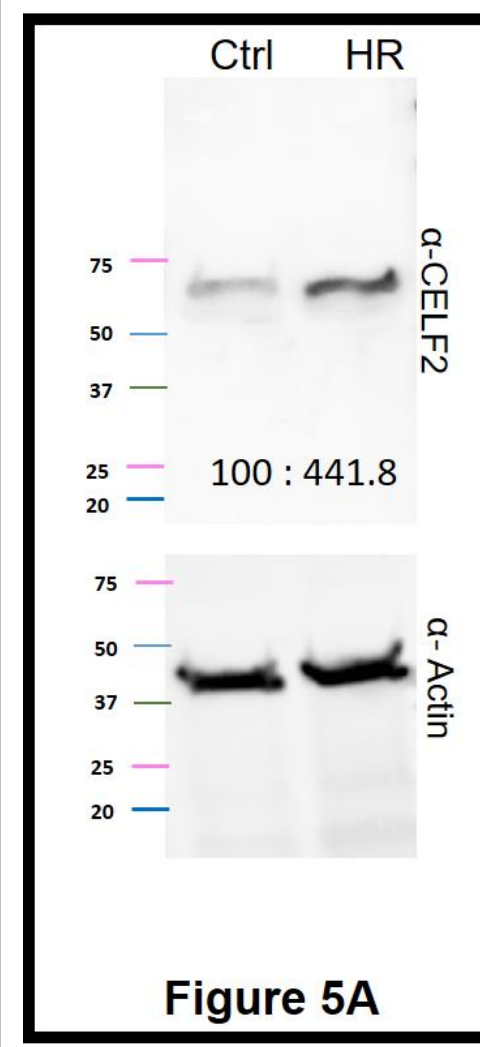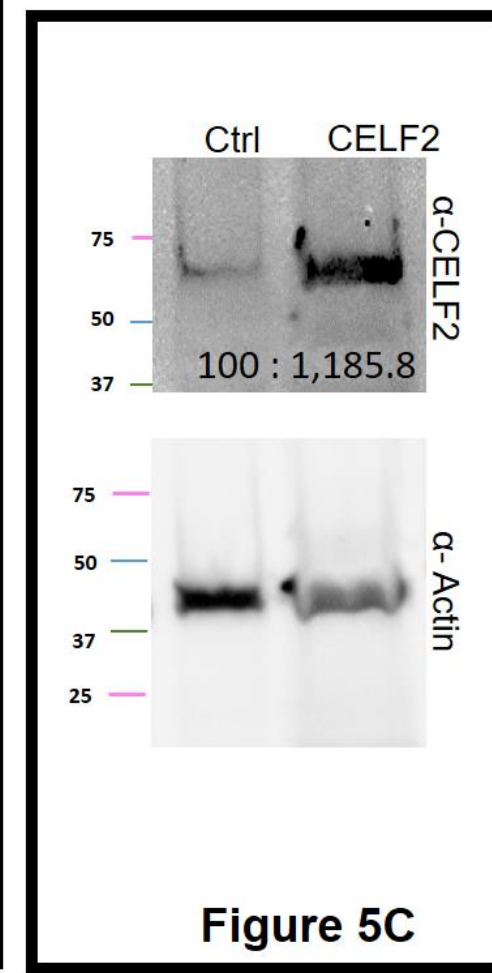

Supplement: Supplementary file 1 [file cancers-14-04648-s001.zip › supplementary File S1.pdf]
